# Epigenetic Diversity of Clonal White Poplar (*Populus alba* L.) Populations: Could Methylation Support the Success of Vegetative Reproduction Strategy?

**DOI:** 10.1371/journal.pone.0131480

**Published:** 2015-07-06

**Authors:** Francesco Guarino, Angela Cicatelli, Giuseppe Brundu, Berthold Heinze, Stefano Castiglione

**Affiliations:** 1 Dipartimento di Chimica e Biologia, Università degli Studi di Salerno, Fisciano, Italia; 2 Dipartimento di Agraria, Università degli Studi di Sassari, Sassari, Italia; 3 Department of Forest Genetics, Austrian Federal Research Centre for Forests, Vienna, Austria; University of Milano Bicocca, ITALY

## Abstract

The widespread poplar populations of Sardinia are vegetatively propagated and live in different natural environments forming large monoclonal stands. The main goals of the present study were: i) to investigate/measure the epigenetic diversity of the poplar populations by determining their DNA methylation status; ii) to assess if and how methylation status influences population clustering; iii) to shed light on the changes that occur in the epigenome of ramets of the same poplar clone. To these purposes, 83 white poplar trees were sampled at different locations on the island of Sardinia. Methylation sensitive amplified polymorphism analysis was carried out on the genomic DNA extracted from leaves at the same juvenile stage. The study showed that the genetic biodiversity of poplars is quite limited but it is counterbalanced by epigenetic inter-population molecular variability. The comparison between *Msp*I and *Hpa*II DNA fragmentation profiles revealed that environmental conditions strongly influence hemi-methylation of the inner cytosine. The variable epigenetic status of Sardinian white poplars revealed a decreased number of population clusters. Landscape genetics analyses clearly demonstrated that ramets of the same clone were differentially methylated in relation to their geographic position. Therefore, our data support the notion that studies on plant biodiversity should no longer be restricted to genetic aspects, especially in the case of vegetatively propagated plant species.

## Introduction

The Mediterranean basin is widely recognized as a biodiversity hot-spot. At the same time it is one of the four hot-spots in the world most significantly affected by human activities [1]. Tree genetic biodiversity of Mediterranean forests is greater than those of central European forests. The high level of biodiversity is the result of various factors, including palaeogeographical [2], ecological and historical [3] that can often be traced back to the Mediterranean ice-age refugia [4]. In a previous study, Brundu *et al*. [5] investigated the genetic biodiversity of poplars, a forest tree species living in Sardinia, demonstrating, through nuclear and chloroplastic microsatellites, that the Sardinian white poplar comprises few genets, and their ramets, in some cases, form huge monoclonal stands in large areas of the island. A similar situation was found by Fussi *et al*. [6] in other Mediterranean islands. Poplars are capable of both sexual and vegetative propagation [7], and it is easy to find admixed or hybrid poplar populations widespread over small or large areas, with overlapping ranges of sexually compatible species. The demographic balance between sexual and vegetative recruitment likely has important consequences for the overall genetic structure of plant populations [5, 6, 8]. The genetic biodiversity of a single organism can be assessed by a range of molecular techniques, [*e*.*g*. AFLP (Amplified Fragment Length Polymorphism) [9] and/or SSR (Simple Sequence Repeat markers) [10]]. Until now, such within-species and population diversity has been described upon the basis of underlying variation in DNA sequences. Although DNA sequence determines the phenotype, emerging evidence indicates that genetic mutations are not the only source of phenotypic variations. Plants, for instance, may use epigenetic mechanisms to respond to environmental variations, or to cope with stress [11, 12]. For instance, Ashikawa [13] found methylation polymorphisms among varieties of cultivated rice, and variation in the degree of methylation of a gene among individuals can sometimes lead to novel phenotypes. Moreover, DNA methylation contributes to transcriptional silencing of transposable elements or foreign DNA, thereby helping to maintain genome stability against non-homologous recombination and regulating the transcription of a number of genes [14–16]. DNA methylation involves the transfer of a methyl group (–CH3) from S-adenosyl-L-methionine to the 5-position of the cytosine pyrimidine ring, or to the 6-nitrogen of the adenine purine ring [17]. In plants, cytosine methylation of DNA occurs at symmetric CpG, CpNpG (where N is any nucleotide and p denotes the sugar-phosphate backbone of DNA), and asymmetric CpHpH sites (where H is “not-G”—adenine [A], cytosine [C], or thymine [T]) [14, 18]. Plant DNA is usually highly methylated, to a level of ca. 6%–30% of methylated sites [19], but there are large differences in the level of 5mC among species [20]. Epigenetic regulation plays an important role in genome protection, DNA repair during replication, gene expression modulation and nuclear inheritance via structural remodelling of chromatin. It is also crucial for promoting phenotypic variation in living organisms [13, 15, 21]. Moreover, Latzel *et al*. [22], analysing the epigenetic diversity of Arabidopsis, found a significant positive relationship between epigenetic diversity and total biomass, and they stated that “*within-species differences*, *and thus potentially*, *functional biodiversity*, *can also be created by epigenetic variation*”. Furthermore, these epigenetic modifications were often stably transmitted through further generations [23]. Many molecular methods have been used to detect DNA methylation status and epigenetic stability [23]. In the last decade, next generation sequencing (NGS) approaches have been developed, such as the development of SNP-based markers (Single Nucleotide Polymorphism) that can be applied to epigenetic studies in humans, animals and plants. This approach is used where a reference genome is available, or not [23, 24]. In the latter case, NGS can be used to sequence new genomes via other methods, for example by using pools of bacterial artificial chromosomes (BACs) clones that facilitates fast *de novo* genome assembly, as demonstrated in barley by Wicker *et al*. [25]. Methylation status can be analysed rapidly by means of Methylation Sensitive Amplified Polymorphism (MSAP). This technique is a powerful, yet cost-effective and highly reproducible molecular method based on AFLP technique that can be adapted to the analysis of genome-wide sequence-specific methylation without *a priori* knowledge of the genomic sequence. Since, at present, the *P*. *alba* genomic sequence is only partially available as a reference [26], and full genome sequencing of many ramets is still beyond the capacity of most laboratories, we adopted the MSAP methodology to analyse the widespread clonal white poplar populations of Sardinia with following aims: i) to investigate/measure the epigenetic diversity of the poplar populations by determining their DNA methylation status, ii) to assess, by studying epigenetic diversity, if and how methylation status influences population clustering and iii) to reveal changes that occur in the epigenome of ramets of the same poplar clone living in different natural environments.

## Materials and Methods

All necessary permissions for sampling were obtained from the landowners. Only a few leaves were collected from each tree without causing any damage. White poplar is neither an endangered nor protected plant species under national or regional law.

### The species studied and its range in Sardinia

The white poplar is a deciduous tree of medium size, growing up to 20–30 m in height and up to 2 m in diameter (at breast height, DBH), forming a broad rounded crown. It commonly propagates by root suckers from the lateral roots, which can occur as far as 20–30 m from the original trunk, and leads to extensive clonal stands. Cladoptosis—the rooting of detached twigs or branches, for instance after flooding–is another mechanism of clonal propagation used by several poplar species [27]. The research was conducted in the island of Sardinia (Italy), sampling 83 trees, distributed across 26 sites with documented GPS data (S1 Table). Each white poplar spot was formed by hundreds, and in some case thousands of individuals.

From each population, six young leaves of the same age and size and from at least two poplar trees were collected (S1 Fig). To minimize the effects of leaf phenology, circadian cycles and seasonal plant growth, we always collected leaves that were not yet fully expanded, at a similar height and at the same juvenile stage, in a short time period (three days), and in quite similar climatic conditions (no rain, sunshine, temperature, morning, etc.). The leaf collections were carried out following the GPS coordinates recorded by Brundu *et al*. [5], and harvesting leaf material from trees of each new sampling site (S1 Table). The white poplar trees previously investigated by Brundu *et al*. [5] with SSR genetic markers were assigned to a genotype indicated by a letter and a number (*e*.*g*. J1, J9, H22, etc.). Newly collected, or not previously assigned, trees are reported here with the abbreviation NA (Not Assigned).

### DNA extraction and MSAP analysis

Total genomic DNA was isolated from the leaves using the DNeasy Plant Mini Kit (Qiagen, Milano-IT). The MSAP method was applied essentially as described by Cicatelli *et al*. [28]. Briefly, we used the isoschizomers *Hpa*II and *Msp*I (methylation sensitive restriction enzymes) as “frequent cutters” and *Eco*RI as a “rare cutter” enzyme (restriction enzyme source: Fermentas, Milano-IT). The restriction enzyme behaviour is reported in Table 1. Two sets of digestion/ligation reactions were carried out simultaneously. In each reaction, 200 ng of genomic DNA were digested for 6 h at 37°C with 5U of each enzyme in 40 μL of digestion buffer (Fermentas). DNA fragments were ligated to *Hpa*II/*Msp*I (5 pmol) and *Eco*RI adapters (0.5 pmol) by the addition of 10 μL of a mix containing 1X ligation buffer and 1.0 U T4 DNA ligase (Fermentas). The mixture was incubated at 22°C for 3 h. The digestion and ligation reactions were stopped by incubating at 65°C for 10 min. The ligation mixture was used as a template for pre-selective amplification with *Eco*+1 and *Msp/Hpa*+0 primers (S2 Table). The PCR reaction was performed under the following conditions: 2 min at 94°C, 20 cycles of denaturation at 94°C for 1 min, annealing at 56°C for 1 min, extension at 72°C for 2 min, and a final elongation step at 72°C for 7 min. The amplification products were diluted 1:20 with sterile distilled water, and a selective PCR was carried out using different primer combinations obtained with *Eco*RI (labelled primers) and *Hpa*II-*Msp*I selective primers, with different selective bases (S2 Table). PCR reaction conditions were: 2 min at 94°C, 1 cycle at 94°C for 30 s, 65°C for 30 s and at 72°C for 1 min. During the initial 13 cycles, the annealing temperature of 65°C was lowered by 0.5°C each cycle, followed by 23 cycles at 94°C for 30 s, 56°C for 30 s and 72°C for 1 min, with a final extension at 72°C for 5 min. PCR products were separated on ABI Prism 310 Genetic Analyzer (Applied Biosystems, Milano, IT), adding 500 μL of GeneScan 500 Rox Size standard (Applied Biosystems, Milano, IT) as internal size standard. MSAP bands (raw data) were analysed by GeneMapper V. 3.7 (Applied Biosystems, Milano, IT). Fragment profiles were transformed into a binary character matrix (S3 and S4 Tables), using 0 or 1 to define the absence or the presence of a specific DNA band, respectively. The MSAP patterns were obtained from the comparison of *Eco*RI-*Hpa*II and *Eco*RI-*Msp*I fragmentation profiles processed independently. By comparing the two profiles, we were able to define the kind of DNA methylation (*e*.*g*. double strand methylation of inner or outer cytosines, hemi-methylation, etc.).

**Table 1:** Restriction enzyme behaviour: *Msp*I and *Hpa*II sensitivity to methylation at cytosines within their recognition target.

*Hpa*II	*Msp*I	Methylation status
1	1	Hemi-methylation of inner cytosine
1	0	Hemi-methylation of outer cytosine
0	1	Double strand methylation of inner cytosine
0	0	Double strand methylation of inner and outer cytosine or absence of target.

Restriction enzyme behaviour in function of full and/or double strand (or hemi-) methylation of inner and/or outer cytosine, 1 indicates the presence of fragment while 0 the absence.

### Biostatistical analyses

In order to determine the DNA methylation status of the Sardinian white poplar populations, biostatistical analyses were carried out by analysing *Eco*RI-*Msp*I and *Eco*RI-*Hpa*II fragmentation profiles separately and together. DNA fragmentation with *Msp*I and *Hpa*II, give different information about DNA methylation status (Table 1). In order to compare the *Msp*I and *Hpa*II data sets, different biodiversity indices [number of bands, number of bands with frequency > 5%, number of private bands, number of locally common bands (frequency > 5%) found in < 25% and < 50% of the populations, mean expected heterozygosity (He), +/- standard error of the mean (SEM)] were estimated using the freely available Arlequin [29] and GeneAlex software packages [30, 31]. In addition, molecular variance analysis (AMOVA) was performed to estimate inter- and intra- population diversity using 9999 permutations of the F*st* value following the methods of Michalakis and Excoffier [32], Peakall *et al*. [33] and Excoffier *et al*. [31]. The matrices were elaborated using Jaccard’s similarity coefficient [34]. On the similarity matrix, a cluster analysis was performed by means of the Unweighted Pair Group Mean with Arithmetical Averages (UPGMA) method using NTSYS-PC (Numerical Taxonomy System, version 2.1 software -http://www.exetersoftware.com/cat/ntsyspc/ntsyspc.html).

In order to determine whether different methylation levels reflect a different population structure, the Structure ver. 2.2 software [35] was used to define the optimal number of clusters and to infer the population structure using *Msp*I and *Hpa*II profiles. The number of populations (K) was estimated by performing 10 runs for each population, from K = 1 to K = 10. Each run consisted of 100,000 MCMC (Markov Chain Monte Carlo) permutations with a burn-period of 10,000, assuming no *a priori* information on population affiliation, the admixture and correlated allele frequencies methods. The optimal population structure was estimated using the method of Evanno *et al*. [36] with 20 independent runs for each K-value.

Landscape analyses were performed on genetic and epigenetic MSAP data in a Bayesian framework using the Geneland R package [37]. Recently, Guillot *et al*. [38] have described a spatial statistical model and used the MCMC technique to estimate the number of populations, assigning individuals to populations of origin and mapping the borders among populations. The populations are assumed to be spatially organized through the coloured Poisson-Voronoi tessellation [39]. Inference was performed via simulation of the posterior distribution of parameters by MCMC using the same parameters as those used in the Structure analyses.

The *msap* package, also available in R environment, allowed us to analyse MSAP data and to assess differences in the *Msp*I and *Hpa*II profiles among groups of samples [40]. On the basis of the presence/absence matrix of both enzymatic reactions, the methylation status of each locus (5′-CCGG target) was assessed: the presence of both *Eco*RI–*Hpa*II and *Eco*RI–*Msp*I fragments (pattern 1/1) denoted a hemi-methylated status; the presence of only one of the *Eco*RI–*Hpa*II or *Eco*RI–*Msp*I fragments represented methylated status (hemi-methylated of the outer C methylation or double strand methylation of the inner cytosine, respectively); the absence of both *Eco*RI–*Hpa*II and *Eco*RI–*Ms*pI fragments (0/0) denoted double strand methylation of inner and outer cytosines or absence of the target sequence (Table 1). Significant differences between relative CG and CNG methylation levels and between relative total methylation and non-methylation levels were estimated by a Wilcoxon rank sum test within each population. The relative CG, CNG methylation and no methylation levels were examined by a Kruskal–Wallis H test. Shannon’s diversity index (I) was calculated to assess the epigenetic diversity (H) of the poplar populations.

## Results

The white poplar population of Sardinia is characterized by a clonal genetic structure. Brundu *et al*. [5], demonstrated by means of chloroplast and nuclear SSRs, that the white poplar populations of Sardinia have three prevalent haplotypes (J, H and L) and only about 20 different genotypes. The presence of large genetically uniform spots of Sardinian white poplar, formed by several ramets, was confirmed by the Bayesian approach used here. In fact, all Sardinian samples (16 to 41 in S2 Fig) had a high probability to belong to the same group. Given the clonal propagation strategy adopted by the Sardinian white poplar, this population is suitable and very useful to estimate the DNA epigenetic status. To achieve the aims of the present study, the MSAP analysis was performed on 83 white poplar trees, collected at different sites across the island of Sardinia.

### Biodiversity and population structure using *Msp*I and *Hpa*II profiles

As described in the Material and Methods, white poplars are widespread in Sardinia, but the population is fragmented. All poplars growing at the same site showed a quite similar *Msp*I fragmentation pattern, even when separated by more than 300 m and interrupted or separated by artificial barriers such as houses, roads, bridges.

Environmental effects on DNA hemi-methylation status was tested by analysing *Msp*I and *Hpa*II fragmentation profiles. By comparing the two profiles, we could evaluate how methylation status influences sample clustering and epigenetic diversity. This is fundamental to understand how environmental conditions can modify DNA methylation in terms of full (double strand), hemi- (single strand), inner- or outer- cytosine methylation. The results of the main indices used to study biodiversity (private and common bands, and the expected heterozygosity) are reported in Tables 2 and 3. With the term “populations” we denote the geographical spots, identified at a distance of about 300 m, or physical barriers sufficient to discriminate different spots. The populations investigated were not exactly the same size because the trees were sampled in relation to population extension.

**Table 2:** Genetic indices calculated for the *Msp*I pattern.

Population	No. Bands	No. Bands (Frequency > 5%)	No. Private Bands	No. Comm Bands (freq. < 25%)	No. Comm Bands (freq. < 50%)	Mean He	SEM
**Pop1**	**119**	**119**	**2**	**2**	**10**	**0**	**0**
**Pop2**	**112**	**112**	**0**	**0**	**8**	**0**	**0**
**Pop3**	**123**	**123**	**0**	**2**	**9**	**0**	**0**
**Pop4**	**123**	**123**	**0**	**2**	**9**	**0**	**0**
**Pop5**	**123**	**123**	**0**	**2**	**9**	**0**	**0**
**Pop6**	**123**	**123**	**0**	**2**	**9**	**0**	**0**
**Pop7**	**114**	**114**	**0**	**0**	**3**	**0**	**0**
**Pop8**	**115**	**115**	**0**	**0**	**4**	**0**	**0**
**Pop9**	**120**	**120**	**0**	**1**	**13**	**0**	**0**
**Pop10**	**116**	**116**	**0**	**1**	**8**	**0**	**0**
**Pop11**	**119**	**119**	**0**	**1**	**9**	**0**	**0**
**Pop12**	**119**	**119**	**0**	**0**	**7**	**0**	**0**
**Pop13**	**117**	**117**	**0**	**0**	**8**	**0,003**	**0,003**
**Pop14**	**118**	**118**	**0**	**0**	**4**	**0**	**0**
**Pop15**	**120**	**120**	**0**	**0**	**5**	**0**	**0**
**Pop16**	**123**	**123**	**1**	**1**	**7**	**0,051**	**0,011**
**Pop17**	**110**	**110**	**0**	**4**	**9**	**0,045**	**0,01**
**Pop18**	**110**	**110**	**0**	**4**	**6**	**0,006**	**0,004**
**Pop19**	**111**	**111**	**0**	**4**	**6**	**0,003**	**0,003**
**Pop20**	**111**	**111**	**0**	**4**	**6**	**0**	**0**
**Pop21**	**110**	**110**	**0**	**7**	**9**	**0,029**	**0,009**
**Pop22**	**110**	**110**	**1**	**5**	**9**	**0,025**	**0,008**
**Pop23**	**106**	**106**	**0**	**5**	**7**	**0,019**	**0,007**
**Pop24**	**108**	**108**	**0**	**5**	**9**	**0,014**	**0,006**
**Pop25**	**112**	**112**	**0**	**1**	**9**	**0**	**0**
**Pop26**	**112**	**112**	**0**	**1**	**9**	**0,002**	**0,002**

Abbreviations: No, number; Mean He, mean expected heterozygosity (He) with its standard errors (SEM).

**Table 3:** Genetic indices calculated for the *HpaII* pattern.

Population	No. Bands	No. Bands (Frequency > 5%)	No. Private Bands	No. Comm Bands (freq. < 25%)	No. Comm Bands (freq. < 50%)	Mean He	SEM
**Pop1**	**72**	**72**	**0**	**1**	**5**	**0**	**0**
**Pop2**	**95**	**95**	**0**	**1**	**5**	**0,099**	**0,015**
**Pop3**	**90**	**90**	**0**	**1**	**5**	**0,073**	**0,013**
**Pop4**	**95**	**95**	**0**	**1**	**5**	**0,101**	**0,015**
**Pop5**	**87**	**87**	**0**	**0**	**2**	**0,031**	**0,009**
**Pop6**	**94**	**94**	**0**	**0**	**4**	**0,014**	**0,006**
**Pop7**	**113**	**113**	**0**	**1**	**18**	**0,074**	**0,013**
**Pop8**	**97**	**97**	**0**	**0**	**15**	**0,006**	**0,004**
**Pop9**	**100**	**100**	**0**	**1**	**8**	**0,034**	**0,009**
**Pop10**	**97**	**97**	**0**	**0**	**15**	**0,024**	**0,008**
**Pop11**	**98**	**98**	**0**	**0**	**15**	**0**	**0**
**Pop12**	**106**	**106**	**0**	**1**	**18**	**0**	**0**
**Pop13**	**96**	**96**	**0**	**0**	**14**	**0,003**	**0,003**
**Pop14**	**115**	**115**	**0**	**0**	**18**	**0**	**0**
**Pop15**	**107**	**107**	**0**	**0**	**19**	**0**	**0**
**Pop16**	**107**	**107**	**0**	**0**	**17**	**0,083**	**0,014**
**Pop17**	**97**	**97**	**0**	**0**	**11**	**0,061**	**0,012**
**Pop18**	**109**	**109**	**1**	**1**	**5**	**0,092**	**0,014**
**Pop19**	**105**	**105**	**0**	**2**	**10**	**0,08**	**0,014**
**Pop20**	**106**	**106**	**0**	**2**	**10**	**0,083**	**0,014**
**Pop21**	**109**	**109**	**0**	**1**	**8**	**0,209**	**0,018**
**Pop22**	**97**	**97**	**0**	**1**	**6**	**0,053**	**0,011**
**Pop23**	**103**	**103**	**1**	**0**	**7**	**0,07**	**0,013**
**Pop24**	**110**	**110**	**0**	**3**	**15**	**0,076**	**0,013**
**Pop25**	**118**	**118**	**0**	**1**	**12**	**0,067**	**0,012**
**Pop26**	**100**	**100**	**0**	**0**	**10**	**0**	**0**

Abbreviations: No, number; Mean He, mean expected heterozygosity (He) with its standard errors (SEM).

The results obtained from the *Msp*I and *Hpa*II MSAP profiles were quite similar (Tables 2 and 3). For example, the number of private bands (which appear only in few populations) in each population was zero, for both molecular profiles. The inter- and intra-population molecular variance was 15% and 85%, respectively, suggesting that the methylation status, revealed by *Eco*RI-*Msp*I digestion, was homogeneous within all the populations studied, while, using *Eco*RI-*Hpa*II digestion, the molecular variance among populations was similar (52% and 48%, respectively). Comparing the *Msp*I and *Hpa*II AMOVA results, it is clear that outer cytosine hemi-methylation increased the differentiation within the populations, thereby reducing the variance among the populations.

### Population structure and genetic similarity analyses

Structure of the Sardinian white poplar populations was analysed with no *a priori* information, using the Structure software [35]. The statistical model described by Evanno *et al*. [36] showed a clear peak (Δ*K* = 14.008) at the *K* value of 3 for the *Msp*I and 2 for *Hpa*II data (Δ*K* = 493.613).

**Fig 1:**
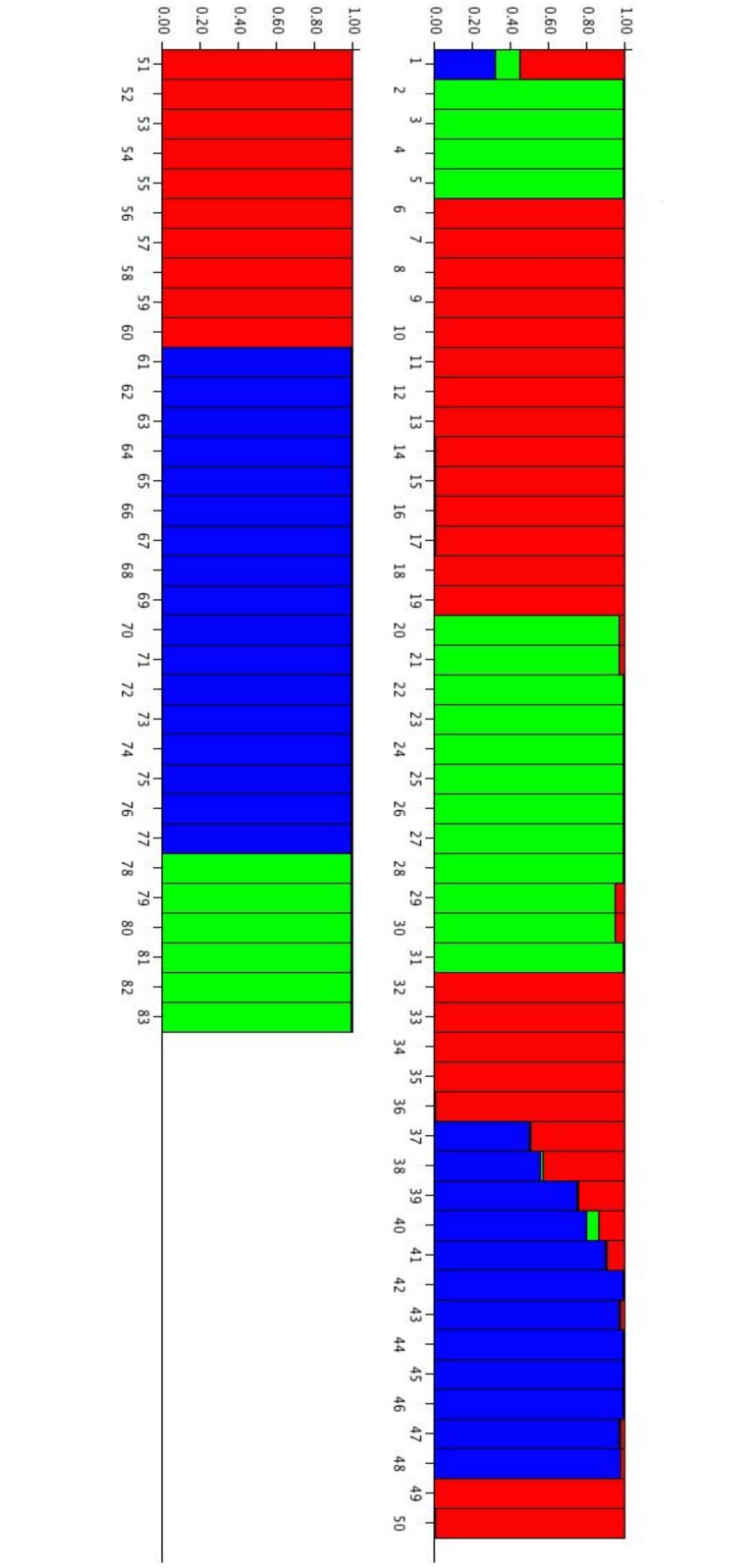
Bar plot of estimated membership probability (*Q*) for K = 3 for the *Msp*I data of Sardinian white poplars. Sample numbers are indicated on the X axis. The estimated membership probability (*Q*) for K = 3 are represented on the Y axis.

**Fig 2:**
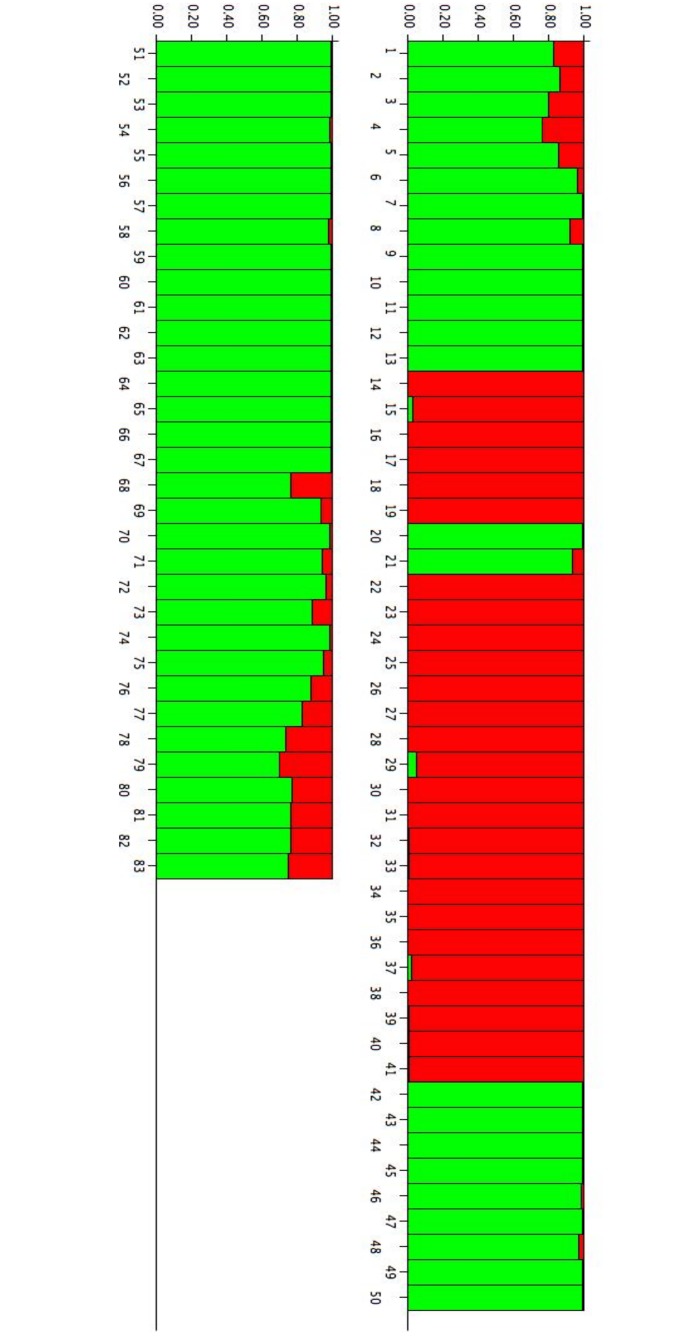
Bar plot of estimated membership probability (*Q*) for K = 2 for the *HpaII* data of Sardinian white poplars. Sample numbers are indicated on the X axis. The estimated membership probability (*Q*) for K = 2 are represented on the Y axis.

Each tree is represented by a vertical bar and classified on the basis of its estimated membership probability in each cluster (*Q*) as reported in Figs 1 and 2. The analyses of the population structure helped us to understand how it was influenced by the methylation status; considering the *Msp*I or *Hpa*II profiles, we obtained different results in terms of K and of membership, due to differences in cytosine methylation.

The K value was also estimated using the Geneland package implemented in R [37, 38]. It allows the estimation of the optimal number of populations in a set, and the analysis of genetic data in relation to geographic coordinates (landscape genetics). Molecular data were processed using the same parameters as those used in Structure. The estimated K for the *Msp*I and *Hpa*II data were 2 and 1, respectively.

**Fig 3:**
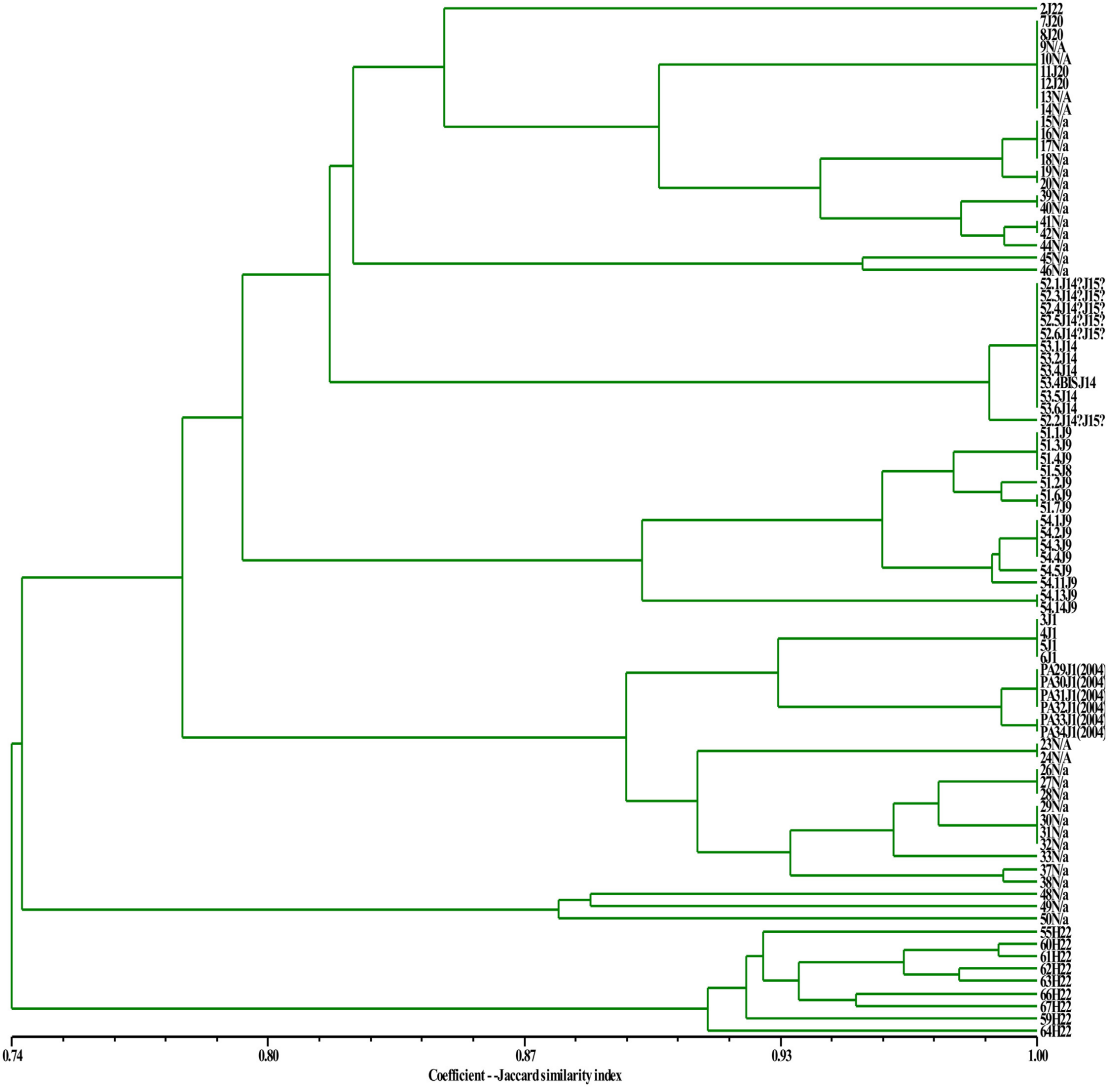
UPGMA dendrogram based on *Msp*I profiles obtained from Sardinian white poplars. The Jaccard similarity index is indicated on the X axis. The sample names with their previously assigned (or not) genotype are reported on the Y axis.

**Fig 4:**
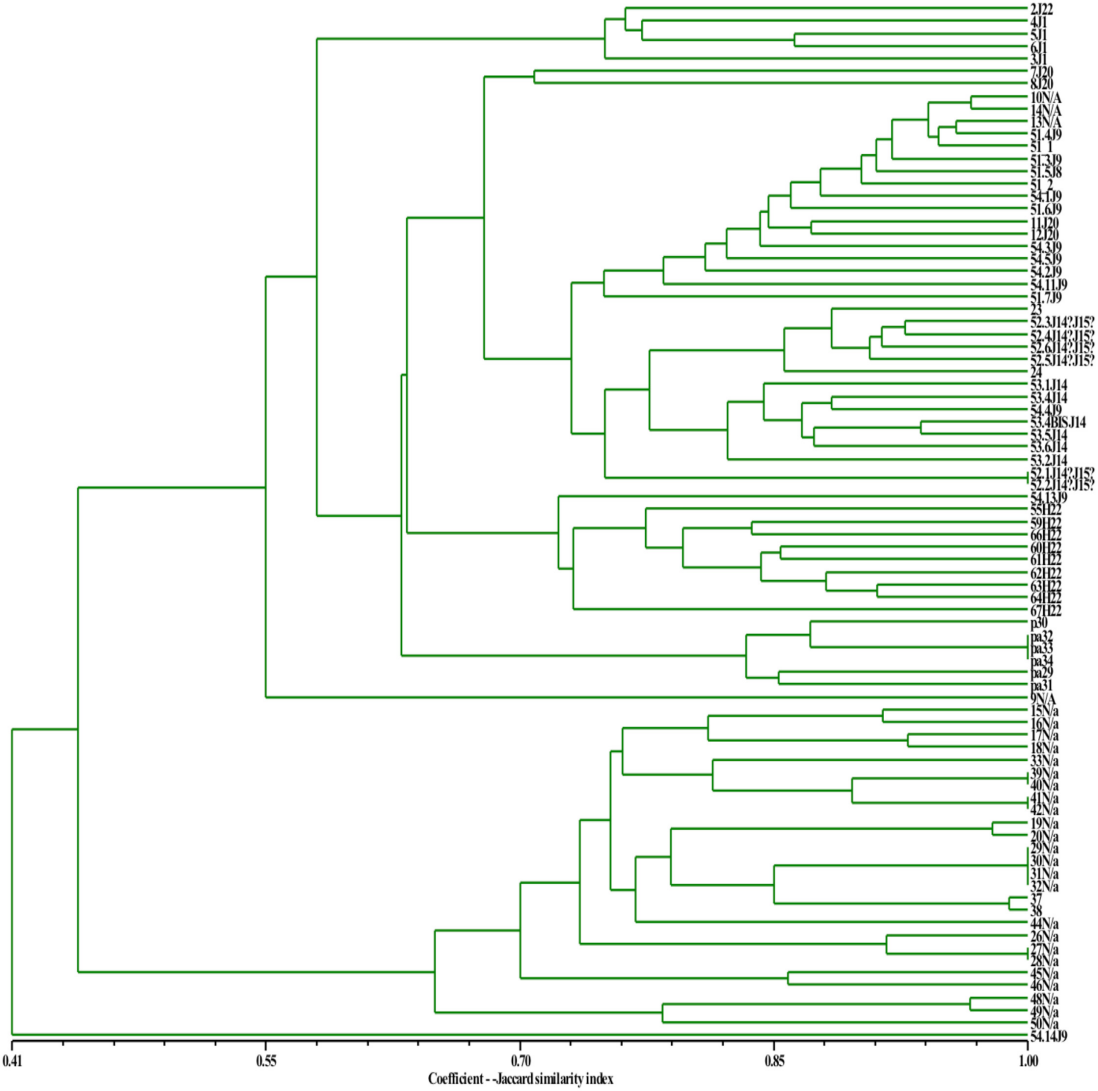
UPGMA dendrogram based on *Hpa*II profile obtained from Sardinian white poplars. The Jaccard similarity index is indicated on the X axis. The sample names with their previously assigned (or not) genotype are reported on the Y axis.

In order to understand how methylation status influences the clustering of the sample and their membership, the Jaccard similarity index was calculated for *Msp*I and *Hpa*II profiles (Figs 3 and 4, respectively). Results, showed a high overall similarity in the Sardinian white poplars (Fig 3). Many trees showed the same methylation status with regard to the methylation of double strand cytosine and hemi-methylation of inner cytosine, whilst the dendrogram constructed using *Hpa*II data showed a lower Jaccard similarity index compared to *Msp*I, due to a larger epigenetic diversity revealed by the former digestion (Fig 4).

To quantify the differences between the epigenetic distances calculated on the basis of the *Msp*I and *Hpa*II data, and to elucidate how methylation status influences the membership in clusters, the *Hpa*II epigenetic distance matrix was subtracted from that of *Msp*I. Therefore, it is possible to have cases where the two distances are roughly similar (resulting in values around zero). However, in other cases, where the *Msp*I distances are greater than the *Hpa*II distances, it results in somewhat smaller values for the subtractions, compared to the unmodified *Msp*I distances. If the opposite is true (*Hpa*II distances greater than *Msp*I), negative values are present. In our study, the *Hpa*II distance was almost always greater than the *Msp*I distance (S5 Table), confirming an important role for DNA hemi-methylation in the plant epigenome, since the differences between individuals are enhanced.

### Estimation of methylation level

In order to estimate the DNA methylation level, MSAP fragments were processed with the *msap* R package, and *a priori* information regarding the number of populations was used. In this case, the population number was set to 3 because it was the optimal population number obtained from Structure analyses of *Msp*I profiles. Moreover, the Shannon diversity index, independently calculated for *Hpa*II and *Msp*I profiles, was 0.592 and 0.139, respectively. A Wilcoxon rank sum test with continuity correction resulted significant (p value = 0.003). The number of the polymorphic loci sensitive to methylation, revealed by *Msp*I digestion, was quite low (12%).

**Fig 5:**
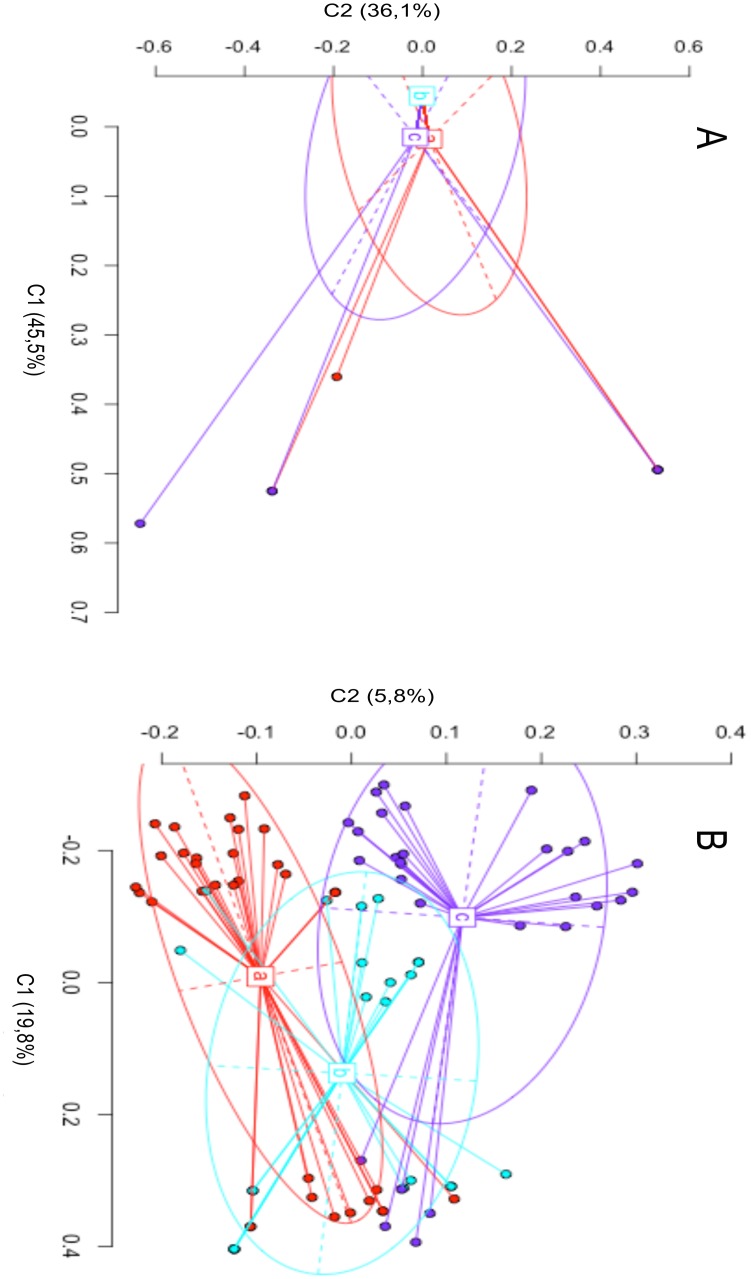
Representation of Principal Coordinate Analysis (PCoA) for epigenetic differentiation among groups. Different colours represent different populations, called a, b, c. The a, b, c, symbols indicate the position of the genetic centroids. Ellipses represent the average dispersion of those individual data points around their centre. The long axis of the ellipse shows the direction of maximum dispersion and the short axis, the direction of minimum dispersion. Fig 5A and 5B show *Msp*I and *Hpa*II results, respectively.

The Principal Coordinate Analysis (PCoA—Fig 5), calculated using the *Msp*I and *Hpa*II profiles (Fig 5A and 5B), showed how epigenetic diversity, regarding the hemi-methylation of outer cytosine, was larger than that revealed by the *Msp*I profile (Table 4). The *Msp*I PCoA showed that the three estimated populations were intersected and shared a large part of the ellipse areas (Fig 5B); on the contrary, the *Hpa*II PCoA showed three clearly distinct populations with partial overlapping. The length of the major and minor axes of the ellipses, representing the dispersion degree of the trees, showed that the dispersion was lower in the case of *Hpa*II data when compared to those produced by *Msp*I digestion. This finding was further confirmed by C1 and C2 values, representing the percentage of the explained variance, which were much smaller in the case of *Hpa*II PCoA.

**Table 4:** Report of methylation levels.

*Hpa*II	*Msp*I	Methylation status	Group a	Group b	Group c
1	1	Hemi-methylation of inner cytosine	**0.443**	**0.503**	**0.345**
1	0	Hemi-methylation of outer cytosine	**0.129**	**0.099**	**0.197**
0	1	Double strand methylation of inner cytosine	**0.289**	**0.234**	**0.308**
0	0	Double strand methylation of inner and outer cytosine or absence of target.	**0.139**	**0.164**	**0.149**

The presence of the fragment is indicated with **+**, while the absence with–. Groups a, b and c are the three clusters obtained from Structure analyses.

## Discussion

Our study of the white poplar populations of Sardinia is one of the first epigenetic diversity studies of a forest tree species. The basic pattern of population differentiations of the Sardinian white poplar was previously studied by Brundu *et al*. [5] using the SSR molecular technique. Those analyses showed the prevalence of vegetative reproduction and, at the same time, demonstrated that Sardinian white poplar populations are genetically isolated from those of the Italian mainland, in particular from those thriving in the Ticino river park (northern Italy). This result was confirmed here by Bayesian statistical analyses. Structure analysis, in fact, highlighted that the Sardinian white poplar populations are separated from those living on the European mainland. Furthermore, Fussi *et al*. [6] and Santos-del-Blanco *et al*. [41] observed a similar reproduction strategy for the white poplar and its hybrid [*P*. *x canescens* (Aiton) Sm] in the Malta archipelago and in Spain, respectively. In the first case, 28 white poplars, sampled throughout the archipelago of Malta, belonged to a single genet; in addition, the cpSSR analysis demonstrated that the Maltese poplars are genetically related to those thriving on the Italian mainland. Therefore, Fussi *et al*. [6] suggested that clonal propagation, very frequent in many poplar species (in particular of those belonging to the section *Populus*), is the only propagation strategy that occurs for white poplars in Malta. Although it is known that DNA sequence determines the phenotype, emerging evidence indicates that in plants, for instance, epigenetic mechanisms may be involved in the response to environmental variations, stimuli and/or stress. DNA methylation changes, in fact, allow the adaptation to the environment via short- to medium- term responses [42], if compared to classical genetic evolution [43]. Latzel *et al*. [22] recently demonstrated that epigenetic diversity increases the productivity and stability of plant populations. Given the previous genetic data, and the concept of epigenetic diversity introduced by Latzel *et al*. [22], we decided to investigate further the Sardinian white poplar populations. These populations represent an ideal model in which to assess the epigenetic diversity in a widespread forest tree species. In our opinion, they were suitable candidates to assess whether, and how, different environmental conditions could affect DNA methylation status, which, in turn, modulates gene expression [5].

To these purposes, the white poplar populations were analysed through the whole genome ‘excerpt’ molecular technique (MSAP) that allows the estimation of epigenetic diversity and of DNA methylation status. Both *Msp*I and *Hpa*II profiles were analysed by molecular variance analysis (AMOVA) to determine whether the Sardinian white poplar populations could be assigned to a single group. The results concerning the partitioning of variance components were different for *Msp*I and *Hpa*II. In fact, the molecular intra-population variance component of the *Msp*I profile was found to be about one third, compared to *Hpa*II profile. Regarding the molecular inter-population variance, it could be said that the *Hpa*II profiles are more homogeneous than those of *Msp*I. This supports the hypothesis that DNA hemi-methylation confers stability to the epigenome among the populations analysed. For both profiles, the hypothesis test of the AMOVA allowed us to reject the null hypothesis (no differentiation either within or among populations).

The results of the statistical analysis conducted on *Msp*I and *Hpa*II can help to understand if, and how methylation status influences the Sardinia white poplar population clustering. The results defined three and two population clusters in the *Msp*I and *Hpa*II profiles, respectively. Therefore, the memberships of individual trees in clusters were different for the *Msp*I and *Hpa*II profiles, suggesting that methylation status modifies the population structure, thereby altering the epigenetic diversity of the white poplar populations.

The *Msp*I and *Hpa*II patterns subjected to the landscape genetic analyses (Geneland) showed that K equal 2 was the most likely number for the *Msp*I profile, while equal 1 for *Hpa*II. Geneland also showed that locations in the simulations lead to a change of K value. This was probably due to the fact that the populations are geographically closer, while in the Structure analyses, they are sufficiently different to join a different cluster. Geneland confirmed that the K value also decreased when the *Hpa*II profiles were analysed. The UPGMA statistic, which is based on a substantially different mathematical approach from that used by Structure and Geneland, confirmed not only the limited diversity of the Sardinian poplars, but also that DNA methylation status modified the clustering and the membership of the populations. Even though the K value decreased from *Msp*I (3) to *Hpa*II (2), it does not mean that the diversity decreased. In fact, the epigenetic diversity revealed by *Hpa*II was more pronounced than that revealed by *Msp*I. This higher inter-population diversity did not allow us to separate the samples into more clusters. The differences between *Msp*I and *Hpa*II profiles were quantified by subtracting the genetic distance, calculated on *Hpa*II data, from that calculated on the *Msp*I profile. The values resulted highly negative because the *Hpa*II genetic distances were higher than those of *Msp*I data. The *Hpa*II profile reacts to the DNA hemi-methylation status of the inner and outer cytosines, whilst *Msp*I only to the hemi-methylation of the inner cytosine; therefore, this large difference could be due to the hemi-methylation of the outer cytosine. Moreover, *msap* analyses suggest that the highest degree of methylation occurred in the inner cytosine, in the form of hemi-methylation. Therefore, the large difference in terms of genetic distance between the *Hpa*II and *Msp*I profiles could be due to less frequent methylation of outer cytosines. The *msap* analyses were performed considering the K value equal 3 (output value of *Msp*I Structure analysis) in order to emphasize the differences between *Msp*I and *Hpa*II profiles. The PCoA outputs related to the *Hpa*II profiles showed three ellipses that gather all the white poplar trees resulting significantly different (p <0.001), while they were not significantly different when the *Msp*I profiles were considered. DNA methylation levels are commonly high in many but not all plant species. Kovarik *et al*. [44] investigated several angiosperm species using restriction enzymes that were differently susceptible to methylation and revealed that they were characterized by different degrees of DNA methylation. Moreover, Hauben *et al*. [45] observed different MSAP profiles in cotyledons and leaves of canola, and Teyssier *et al*. [46] demonstrated that the DNA methylation level varied during fruit ripening in the mature leaves and pericarp of the tomato.

Our study is in agreement with that of Ma *et al*. [47]. This study showed that hemi-methylation was the more frequent event; however, the same authors previously demonstrated that the methylation of inner cytosines, analysed by MSAP, was slightly higher than hemi-methylation in Chinese white poplar [48]. This discrepancy may be due to tissue-specific DNA methylation; the authors investigated leaf DNA in the first case [48] and wood in the latter [47]. They analysed wood in order to reduce variables associated with the analysis of organs that are formed of different tissues. One of the aims of our study, on the contrary, was to assess environmental effects on the leaf epigenome in the white poplar populations of Sardinia, which are characterized by a very low genetic biodiversity [5]. Since leaf is a photosynthetic plant organ with additional perception functions (*e*.*g*. light, altitude, temperature, atmospheric pollutants, etc.), it can provide information about epigenetic modification and adaptation induced by short- and/or medium-term environmental changes. Only few reports have shown epigenome alterations in response to different environmental conditions. For instance, one such study focused on mangroves growing at riversides compared to those grown in salt marshes [49]. Moreover, these epigenetic modifications were often stably transmitted through further generations [23]; in fact, plants being sessile organisms, react to different environmental stimuli through epigenetic modifications [28, 50–52] that are storable in a kind of ‘plant memory’, transmissible to the progeny [53] and able to regulate gene expression [53–55]. The mechanisms of epigenetic memory in relation to environmental stresses are not yet fully understood [56]. Thellier and Lüttge [53] developed a hypothesis regarding stress response by plants through ‘methylation episodes’ that might lead to higher overall levels of methylation, thus constituting a ‘storage mechanism’. According to their hypothesis, epigenetic changes can be triggered by external stimuli as demonstrated by several authors [55, 57–59]. These changes may involve the production of chemical signals such as phytohormones, electrical signals and calcium channel activation [60].

To ensure that stress memory is maintained by methylation status, it is necessary that, when the stress is passed, the methylation status does not return to the previous level [55]. An example of the epigenetic stress response and methylation hereditary transmission is that of Suter and Widmer [61], who demonstrated that, in Arabidopsis seedlings, a high salinity stress induced potential heritable phenotypic adaptations regardless of genetic variation. Moreover, Latzel *et al*. [22] reported that methylation status can increase the productivity and stability of plant populations, a condition named ‘phenotypic plasticity’ by other authors [62]. In particular, Arabidopsis populations with dissimilar epigenetic status produced up to 40% more biomass than highly uniform populations. This suggests that it would be appropriate to include epigenetic research in ecological studies in order to quantify the natural epigenetic diversity and test its consequences among many different species. In our study, the altered epigenetic status revealed within the Sardinian white poplar populations modified the number of clusters and the tree memberships. Considering the same clonal populations previously investigated by Brundu *et al*. [5], we verified that the ramets of the same clone showed different methylation status in relation to their geographical position. For instance, each tree of the J9 genotype showed a very similar *Msp*I profile, but this was not the case when the *Hpa*II profile was considered. To our knowledge, this is the first study that demonstrates how poplar trees, genetically identical and living in different sites, have a diverse hemi-methylation status, presumably as a result of different environmental and edaphic conditions. Furthermore, our research showed that methylation status modifies population structure. Although *Msp*I fragmentation is influenced by methylation, the *Msp*I epigenetic profiles provided the same information derived from the previous SSR analysis [5], indicating that the *Msp*I methylation status was only slightly modified by different environmental conditions.

In conclusion, our study revealed that the genetic biodiversity of the Sardinian white poplar is quite limited, but it is compensated by epigenetic inter-population diversity, which possibly allows white poplars to grow in very large areas of the island of Sardinia, supporting the success of their vegetative reproduction strategy. Epigenetic variations are frequent and occur more rapidly in response to environmental stimuli. Furthermore, they are much easier appreciable in populations that propagate predominantly by clonal reproduction, as shown here for the Sardinian white poplar.

## Supporting Information

S1 Fig Example of collected leaves put on graph paper. (TIF)

S2 Fig Bar plot of estimated membership probability (*Q*) for K = 2 of data furnished by Brundu *et al*. [5]. Sample numbers are indicated on the X axis. The estimated membership probability (*Q*) for K = 2 is indicated on the Y axis. Samples 1 to 15 = *P*. *tremula*, Scotland (reference samples); samples 16 to 42 = *P*. *alba*, Sardinia; samples from 43 to 90 = *P*. *alba*, Ticino; samples 91 to 118 = *P*. *alba* Mediterranean basin.
(TIFF)

S1 Table Geographic coordinates of the collected white poplar trees and their assigned (or not) genotype. (PDF)

S2 Table Table of used primers. (XLSX)

S3 Table Raw data of *Eco*RI-*Msp*I digestion. (XLSX)

S4 Table Raw data of *Eco*RI-HpaII digestion. (XLSX)

S5 Table Difference between genetic distance matrices calculated for HpaII and MspI profiles. (XLSX)
