# Supplementary figures and images for: Epigenetic Diversity of Clonal White Poplar (Populus alba L.) Populations: Could Methylation Support the Success of Vegetative Reproduction Strategy?
Source: PLoS One. 2015 Jul 6;10(7):e0131480. doi: 10.1371/journal.pone.0131480 (PMC4492942; doi:10.1371/journal.pone.0131480)

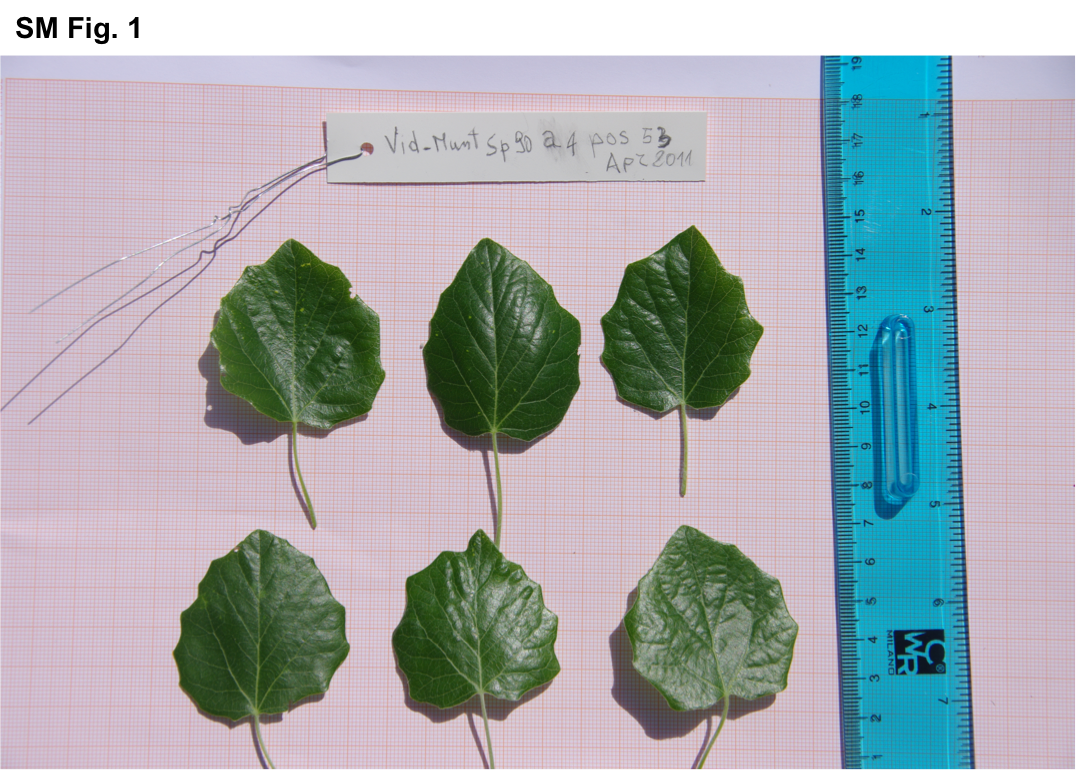

Supplement: S1 Fig — (TIF) [file pone.0131480.s001.tif]

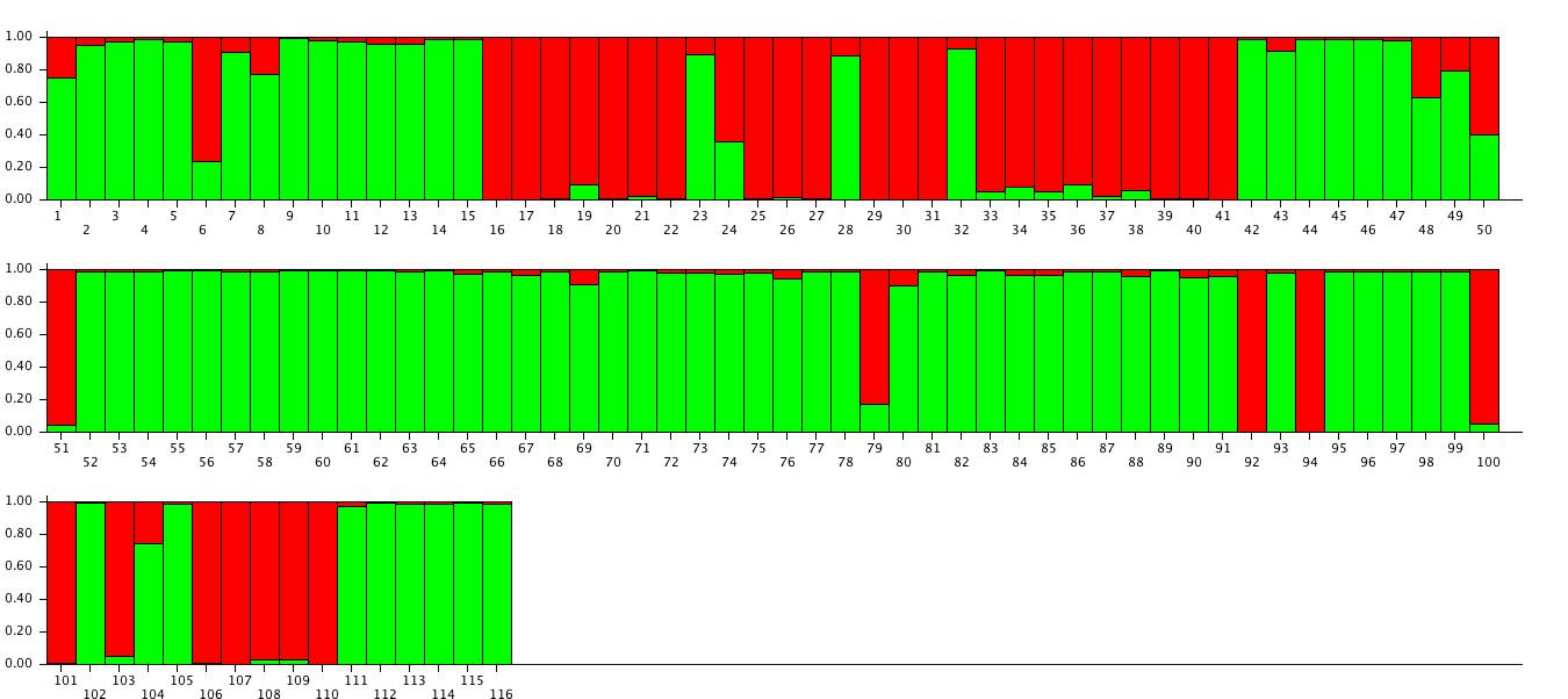

Supplement: S2 Fig — Sample numbers are indicated on the X axis. The estimated membership probability (Q) for K = 2 is indicated on the Y axis. Samples 1 to 15 = P. tremula, Scotland (reference samples); samples 16 to 42 = P. alba, Sardinia; samples from 43 to 90 = P. alba, Ticino; samples 91 to 118 = P. alba Mediterranean basin. (TIFF) [file pone.0131480.s002.tiff]
