# Supplementary material for: Epigenetic Diversity of Clonal White Poplar (Populus alba L.) Populations: Could Methylation Support the Success of Vegetative Reproduction Strategy?
Source: PLoS One. 2015 Jul 6;10(7):e0131480. doi: 10.1371/journal.pone.0131480 (PMC4492942; doi:10.1371/journal.pone.0131480)
Supplement: S1 Table — (PDF) [file pone.0131480.s003.pdf]

Table S1

Sample name	Population	UTME (WGS84)	UTMN (WGS84)	Geographical zone	Altitude (m)	City	Assigned genotype by Brundu et al . (2008)
2J22	Pop1	491648	4520290	32T	39,01	Perfugas	J22
2bisJ22	Pop1	488763	4532421	32T	14,33	Padulo (Badesi)	J22
3J1	Pop2	488758	4532419	32T	14,02	Padulo (Badesi)	J1
4J1	Pop2	488861	4532362	32T	11,28	Padulo (Badesi)	J1
5J1	Pop2	488874	4532372	32T	8,53	Padulo (Badesi)	J1
6J1	Pop2	491208	4528397	32T	9,45	Viddalba	J1
7J20	Pop3	491231	4528417	32T	-3,05	Viddalba	J20
8J20	pop3	494757	4524140	32T	60,05	Fraigata	J20
9N/A	Pop4	494738	4524178	32T	56,39	Fraigata	NOT ASSIGNED
10N/A	Pop4	494455	4523545	32T	44,50	Tiesennari	NOT ASSIGNED
11J20	Pop5	494421	4523564	32T	38,10	Tiesennari	J20
12J20	Pop5	494430	4523391	32T	34,44	La Fraigata	J20
13N/A	Pop6	494395	4523384	32T	34,75	La Fraigata	NOT ASSIGNED
14N/A	Pop6	490202	4519413	32T	52,12	Perfugas	NOT ASSIGNED
15N/a	Pop7	490084	4519471	32T	55,47	Perfugas	NOT ASSIGNED
16N/a	Pop7	490124	4519456	32T	56,69	Perfugas	NOT ASSIGNED
17N/a	Pop7	490180	4519430	32T	54,25	Perfugas	NOT ASSIGNED
18N/a	Pop7	485516	4509927	32T	338,63	Chiaramonti	NOT ASSIGNED
19N/a	Pop8	485538	4509901	32T	335,58	Chiaramonti	NOT ASSIGNED
20N/a	Pop8	465450	4505511	32T	160,00	Scala di Giocca SS	NOT ASSIGNED
23N/A	Pop9	465450	4505511	32T	160,00	Scala di Giocca SS	NOT ASSIGNED
24N/A	Pop9	468836	4504181	32T	160,00	S. Martino SS	NOT ASSIGNED
26N/a	Pop10	468836	4504181	32T	160,00	S. Martino SS	NOT ASSIGNED
27N/a	Pop10	468836	4504181	32T	160,00	S. Martino SS	NOT ASSIGNED
28N/a	Pop10	469044	4503992	32T	160,00	Rio Mascari	NOT ASSIGNED
29N/a	Pop11	469044	4503992	32T	160,00	Rio Mascari	NOT ASSIGNED
30N/a	Pop11	469044	4503992	32T	160,00	Rio Mascari	NOT ASSIGNED
31N/a	Pop11	469044	4503992	32T	160,00	Rio Mascari	NOT ASSIGNED
32N/a	Pop11	469444	4504125	32T	160,00	Rio Mascari	NOT ASSIGNED
33N/a	Pop12	467118	4504478	32T	265,00	Muros	NOT ASSIGNED
33bisN/a	Pop12	467118	4504478	32T	265,00	Muros	NOT ASSIGNED
37N/a	Pop13	473605	4501663	32T	230,00	Saccargia	NOT ASSIGNED
38N/a	Pop13	473605	4501663	32T	230,00	Saccargia	NOT ASSIGNED
39N/a	Pop14	481892	4488131	32T	300,00	Sorgente Malis	NOT ASSIGNED
40N/a	Pop14	481892	4488131	32T	300,00	Sorgente Malis	NOT ASSIGNED
41N/a	Pop15	479097	4486685	32T	459,00	Bonnanaro	NOT ASSIGNED
42N/a	Pop15	478967	4486574	32T	459,00	Bonnanaro	NOT ASSIGNED
44N/a	Pop16	478967	4486574	32T	459,00	Bonnanaro	NOT ASSIGNED
45N/a	Pop16	472486	4422572	32S	13,00	Solarussa	NOT ASSIGNED
46N/a	Pop16	472486	4422572	32S	13,00	Solarussa	NOT ASSIGNED
48N/a	Pop17	472486	4422572	32S	13,00	Solarussa	NOT ASSIGNED
49N/a	Pop17	480169	4399277	32S	300,00	Morgongiori	NOT ASSIGNED
50N/a	Pop17	480169	4399277	32S	300,00	Morgongiori	NOT ASSIGNED
51.1J9	Pop18	480169	4399277	32S	300,00	Morgongiori	J9
51.2J9	Pop18	480169	4399277	32S	300,00	Morgongiori	J9
51.3J9	Pop18	480169	4399277	32S	300,00	Morgongiori	J9
51.4J9	Pop18	480169	4399277	32S	300,00	Morgongiori	J9
51.5J8	Pop18	480169	4399277	32S	300,00	Morgongiori	J9
51.6J9	Pop18	502332	4410703	32S	350,00	Laconi	J9
51.7J9	Pop18	502332	4410703	32S	350,00	Laconi	J9
52.1J14/J15	Pop19	502332	4410703	32S	350,00	Laconi	J14 or J15
52.2J14/J15	Pop19	502332	4410703	32S	350,00	Laconi	J14 or J15
52.3J14/J15	Pop19	502332	4410703	32S	350,00	Laconi	J14 or J15
52.4J14/J15	Pop19	502332	4410703	32S	350,00	Laconi	J14 or J15
52.5J14/J15	Pop19	502332	4410703	32S	350,00	Laconi	J14 or J15
52.6J14/J15	Pop19	494464	4407887	32S	200,00	Senis	J14 or J15
53.1J14	Pop20	494464	4407887	32S	200,00	Senis	J14
53.2J14	Pop20	494464	4407887	32S	200,00	Senis	J14
53.4J14	Pop20	494464	4407887	32S	200,00	Senis	J14
53.4BISJ14	Pop20	494464	4407887	32S	200,00	Senis	J14
53.5J14	Pop20	494464	4407887	32S	200,00	Senis	J14
53.6J14	Pop20	494464	4407887	32S	200,00	Senis	J14
54.1J9	Pop21	488706	4405302	32S	212,00	Escovedu	J9
54.2J9	Pop21	488706	4405302	32S	212,00	Escovedu	J9
54.3J9	Pop21	488706	4405302	32S	212,00	Escovedu	J9
54.4J9	Pop21	488706	4405302	32S	212,00	Escovedu	J9
54.5J9	Pop21	488706	4405302	32S	212,00	Escovedu	J9
54.11J9	Pop21	488706	4405302	32S	212,00	Escovedu	J9
54.13J9	Pop21	488706	4405302	32S	212,00	Escovedu	J9
54.14J9	Pop21	488706	4405302	32S	212,00	Escovedu	J9
55H22	Pop22	490039	4305946	32S	Not available	Torre di Chia	H22
59H22	Pop22	490039	4305946	32S	Not available	Torre di Chia	H22
60H22	Pop23	489442	4306389	32S	Not available	Chia	H22
61H22	Pop23	489442	4306389	32S	Not available	Chia	H22
62H22	Pop23	489442	4306389	32S	Not available	Chia	H22
63H22	Pop23	489442	4306389	32S	Not available	Chia	H22
64H22	Pop23	489442	4306389	32S	Not available	Chia	H22
66H22	Pop24	477782	4315299	32S	Not available	Teulada	H22
67H22	Pop24	477782	4315299	32S	Not available	Teulada	H22
PA29J1	Pop25	460233	4504438	32T	65	Stazione Molafˆ (Riu Mascari)	J1
PA30J1	Pop25	460202	4504434	32T	69	Stazione Molafˆ (Riu Mascari)	J1
PA31J1	Pop25	460112	4504411	32T	62	Stazione Molafˆ (Riu Mascari)	J1
PA32J1	Pop26	459819	4498429	32T	194	N.a S.a de Paulisi (Uri)	J1
PA33J1	Pop26	459832	4498427	32T	195	N.a S.a de Paulisi (Uri)	J1
PA34J1	Pop26	459827	4498411	32T	198	N.a S.a de Paulisi (Uri)	J1
